# Machine learning prediction of PICC-associated thrombotic complications in critically ill patients

**DOI:** 10.1186/s12911-026-03607-w

**Published:** 2026-05-30

**Authors:** Liyan Xia, Hailan Jiang, Yanfei Zhang, Jiahui Guo, Mengying Sun, Haitao Wu, Yang Cui, Yongjie Wang

**Affiliations:** 1 People’s Hospital of Jilin Province, Changchun, China; 2 Liuhe Town Central Health Center, Liuhe, China; 3 China-Japan Union Hospital of Jilin University, Changchun, China; 4 The First Hospital of Jilin University (Bethune First Hospital), Changchun, China

**Keywords:** Peripherally inserted central catheter, Venous thrombosis, Machine learning, Risk prediction, Critical care, Clinical decision support

## Abstract

**Background:**

Peripherally inserted central catheters (PICCs) are widely used vascular access devices in intensive care, yet thrombotic complications remain a significant clinical concern. Traditional risk assessment tools fail to capture the complex, non-linear interactions among thrombosis risk factors in critically ill patients.

**Methods:**

This retrospective cohort study, conducted in adherence to TRIPOD + AI guidelines, developed and compared five machine learning models for predicting PICC-associated thrombotic complications using data from the Medical Information Mart for Intensive Care IV (MIMIC-IV) database. A total of 8,145 PICC insertions from 6,376 patients were analyzed, with 29 predictor variables spanning demographics, coagulation parameters, hematologic indices, renal function, vital signs, comorbidities, and treatment variables. Synthetic Minority Over-sampling Technique (SMOTE) addressed class imbalance (16.88% event rate), applied exclusively to the training set after the train-test split. Hyperparameters were optimized via five-fold cross-validated grid search.

**Results:**

Random Forest achieved the best discriminative performance (AUC-ROC 0.809, 95% CI: 0.780–0.838, bootstrap *n* = 2,000), significantly outperforming all other models on DeLong’s test (all *P* < 0.01); its calibration slope of 1.369 indicated mild underconfidence. Decision curve analysis confirmed net clinical benefit for the three tree-based ensembles across threshold probabilities of approximately 1–50%. Feature importance analysis identified prior thrombosis history as the dominant predictor, followed by coagulation parameters and hematologic indices. Sensitivity analyses using patient-clustered data splitting, multiple imputation (m = 5, Rubin’s rules), first-PICC-only subsets, and a no-prior-thrombosis subgroup confirmed the robustness of the primary findings.

**Conclusions:**

These findings support the development of machine learning-based clinical decision support tools for individualized thrombosis risk stratification in critical care settings. The completed TRIPOD + AI checklist is provided as Additional file 1.

**Supplementary Information:**

The online version contains supplementary material available at 10.1186/s12911-026-03607-w.

## Background

Peripherally inserted central catheters (PICCs) have become indispensable vascular access devices in modern critical care medicine, with millions of insertions performed annually worldwide. These catheters are increasingly utilized for administration of medications including chemotherapy and antibiotics, parenteral nutrition delivery, hemodynamic monitoring, and blood sampling in intensive care unit (ICU) patients. PICCs offer several advantages over traditional central venous catheters, including ease of insertion, reduced immediate complications, and suitability for prolonged use. However, despite their clinical utility, PICC placement carries inherent risks, with thrombotic complications representing one of the most serious adverse events [1, 2].

Catheter-related thrombosis encompasses a spectrum of clinical manifestations ranging from asymptomatic catheter tip thrombosis detected incidentally on imaging to symptomatic deep vein thrombosis (DVT) requiring therapeutic anticoagulation and life-threatening pulmonary embolism (PE) necessitating emergent intervention [3, 4]. These complications result in significant morbidity, including prolonged hospitalization, increased healthcare costs, catheter malfunction requiring replacement, potential long-term sequelae such as post-thrombotic syndrome, and in severe cases, mortality from massive pulmonary embolism [5, 6]. The reported incidence of PICC-associated thrombosis varies considerably, with rates ranging from 3.0% to over 70% depending on patient population and surveillance methods [7]. Evans et al. [8] reported 3.0% in general hospitalized patients, and Meng et al. [9] reported a pooled incidence of 7.0% (95% CI: 4.0–13.0%) in breast cancer patients; substantially higher rates approaching 50% have been documented when asymptomatic thrombosis is detected through systematic ultrasound surveillance in cancer cohorts [2, 4].

The pathophysiology of catheter-related thrombosis involves a complex interplay of multiple factors described by Virchow’s classic triad: vessel wall injury, blood stasis, and hypercoagulability [2, 10]. In critically ill patients receiving PICCs, vessel wall injury occurs during catheter insertion through mechanical trauma to the endothelium and continues through ongoing catheter-related irritation. Blood flow stasis develops around the catheter due to venous obstruction, particularly with larger bore catheters. Hypercoagulability stems from multiple sources including underlying disease states, systemic inflammatory responses, procoagulant effects of certain medications, and hemostatic activation from tissue trauma [10]. Current clinical practice guidelines do not recommend routine thromboprophylaxis for all PICC patients due to bleeding risks and modest overall incidence of symptomatic thrombosis [10]. However, this approach may lead to undertreatment in high-risk patients while risking overtreatment without systematic risk stratification.

Traditional approaches to thrombosis risk assessment have evolved from simple empirical judgment to systematic evaluation tools such as the Caprini score [11]. However, these conventional scoring systems rely on linear point-based calculations that often fail to capture the multifactorial nature of thrombosis pathophysiology in critically ill patients, where risk factors interact dynamically in complex, non-linear ways. Recent advances in machine learning and artificial intelligence have opened promising new avenues for developing more sophisticated predictive models [12, 13]. Unlike traditional statistical approaches limited to linear relationships, machine learning algorithms can identify complex non-linear relationships among numerous variables, handle high-dimensional data, and adapt to heterogeneous patient populations [12–14]. Several recent studies have successfully applied machine learning techniques to predict clinically important outcomes in ICU settings, including early sepsis detection [15] and acute kidney injury prediction [16]. However, the application of machine learning models trained on broad clinical feature sets for predicting PICC-associated thrombotic complications remains limited, with most existing risk prediction efforts relying on traditional regression-based approaches such as nomograms and logistic risk factor analyses [7, 17].

A critical knowledge gap exists regarding whether systematic integration of comprehensive clinical data across multiple domains—including coagulation parameters, hematologic indices, comorbidity profiles, treatment variables, and physiologic measurements—combined with rigorous comparison of multiple machine learning algorithms, can provide clinically actionable risk assessment for critically ill patients receiving PICCs. This study aimed to develop and validate machine learning models for predicting PICC-associated thrombotic complications using extensive clinical data from the MIMIC-IV database, systematically comparing five different algorithms and identifying the most important predictive features.

## Methods

### Reporting guideline

This study was conducted and reported in accordance with the Transparent Reporting of a multivariable prediction model for Individual Prognosis Or Diagnosis plus Artificial Intelligence (TRIPOD + AI) guidelines [18]. The completed TRIPOD + AI checklist is provided as Additional file 1.

### Study design and data source

This retrospective cohort study utilized data from the MIMIC-IV database (version 3.1), a large, publicly available critical care database containing comprehensive de-identified health records from patients admitted to Beth Israel Deaconess Medical Center in Boston, Massachusetts, between 2008 and 2022 [19]. The database includes demographics, vital signs, laboratory test results, medication administration records, procedure documentation, and diagnoses coded using International Classification of Diseases, Ninth and Tenth Revision (ICD-9/ICD-10) systems.

### Study population

The study population comprised all adult patients (≥ 18 years) who underwent PICC insertion during an ICU stay. From 14,595 distinct PICC insertion events, patients were excluded if they had missing values in any of the 29 predefined predictor variables. The final cohort comprised 8,145 PICC insertions (55.8%) from 6,376 unique patients. The 44.2% exclusion rate reflected incomplete data: 35.5% involved missing coagulation parameters (International Normalized Ratio [INR], Prothrombin Time [PT], Partial Thromboplastin Time [PTT]) and 8.7% missing non-coagulation variables, predominantly vital signs. Sensitivity analyses using multiple imputation were conducted to assess the impact of this exclusion (see Sensitivity analyses below).

### Outcome definition

The primary outcome was PICC-associated thrombotic complications, encoded as a binary variable (1 = thrombosis, 0 = no thrombosis). Thrombotic events were identified through ICD-9 and ICD-10 diagnosis codes during the same hospitalization as PICC insertion, targeting upper extremity venous thrombosis, DVT, superficial thrombophlebitis, catheter-related thrombosis, and venous thromboembolism including PE.

### Predictor variables

Based on established thrombosis risk factors [9, 10], 29 predictor variables were extracted across seven clinical domains: (1) Demographics and clinical variables: age, gender, body weight, time from admission to PICC insertion; (2) Coagulation parameters: INR, PT, PTT; (3) Hematologic indices: platelet count, white blood cell (WBC) count, hemoglobin, red blood cell (RBC) count; (4) Renal function: serum creatinine, blood urea nitrogen (BUN); (5) Vital signs: heart rate, mean arterial pressure (MAP), temperature, respiratory rate, oxygen saturation (SpO_2_); (6) Comorbidities: cancer, prior thrombosis, chronic kidney disease (CKD), diabetes mellitus, cardiovascular disease (CVD); (7) Treatment variables: heparin, warfarin, direct oral anticoagulants (DOAC), antiplatelet therapy, mechanical ventilation, vasopressor support. Laboratory values were obtained within 24 h before PICC insertion; vital signs were averaged over the 24-hour pre-insertion period; medications were documented within 48 h before insertion. All 29 variables are routinely collected during ICU care and accessible through standard electronic health record (EHR) systems.

### Statistical analysis and machine learning

Continuous variables underwent z-score standardization. Categorical variables were encoded numerically (male = 1, female = 0 for gender; presence = 1, absence = 0 for comorbidities and treatments). Continuous variables were compared between groups using Welch’s t-test; categorical variables were compared using the chi-square test. It should be noted that because the unit of analysis was PICC insertions rather than unique patients, some patients contributed multiple observations (8,145 insertions from 6,376 patients), which may violate the independence assumption of these tests. The results in Table 1 should therefore be interpreted with this caveat. The dataset was randomly partitioned into training (80%, *n* = 6,516) and testing (20%, *n* = 1,629) sets using stratified sampling to maintain class proportions. Baseline characteristics of the training and test sets were compared to verify balance (Additional file 2). SMOTE was applied exclusively to the training set after the train-test split to address class imbalance, ensuring that no synthetic samples contained information from the test set [20].

Five machine learning algorithms were developed and compared: (1) Logistic Regression (LR) as the linear baseline; (2) Random Forest (RF); (3) Support Vector Machine (SVM) with radial basis function (RBF) kernel; (4) Gradient Boosting (GB); and (5) XGBoost (XGB). These five algorithms were selected to represent a broad spectrum of learning paradigms—linear, ensemble tree-based, and kernel-based methods—providing diverse approaches to capturing the potentially complex relationships in thrombosis prediction. LightGBM was not included as its architecture closely mirrors XGBoost and Gradient Boosting, and preliminary experiments showed no meaningful performance differences. Deep learning approaches (e.g., artificial neural networks) were not included due to the tabular nature of the dataset and the modest sample size, where tree-based ensemble methods typically outperform neural networks and offer superior interpretability [21].

All models were implemented in Python (version 3.8.20) using scikit-learn (version 1.3.2), XGBoost (version 2.1.4), and imbalanced-learn (version 0.12.4). Hyperparameters were optimized through exhaustive grid search with five-fold stratified cross-validation within the training set, using AUC-ROC as the optimization criterion. The grid search spaces and optimal hyperparameters identified for each model are reported in Additional file 3.

### Model evaluation

Model performance was assessed on the independent test set (*n* = 1,629) using AUC-ROC as the primary discriminative metric, with secondary metrics including accuracy, precision (positive predictive value), sensitivity (recall, true positive rate), specificity (true negative rate), and F1-score (harmonic mean of precision and sensitivity). These metrics are defined as: Accuracy = (TP + TN) / (TP + TN + FP + FN); Precision = TP / (TP + FP); Sensitivity = TP / (TP + FN); Specificity = TN / (TN + FP); F1-Score = 2 × (Precision × Sensitivity) / (Precision + Sensitivity), where TP, TN, FP, and FN denote true positives, true negatives, false positives, and false negatives, respectively. The 95% confidence intervals (CIs) for all performance metrics were computed using the bootstrap method with 2,000 resamples of the test set, using the 2.5th and 97.5th percentiles of the bootstrap distribution. Calibration was assessed using calibration plots and quantified by calibration slope, calibration intercept, and Brier score.

Pairwise model comparisons were conducted using DeLong’s test for the equality of two correlated AUC-ROC curves, with the Random Forest model as the reference [22]. Optimal classification thresholds for each model were determined using the Youden index (J = Sensitivity + Specificity − 1), which maximizes the sum of sensitivity and specificity [23].

### Decision curve analysis

Decision curve analysis (DCA) was performed to evaluate the clinical utility of the prediction models by comparing their net benefit against default strategies of treating all patients or treating no patients across a range of threshold probabilities [24]. DCA quantifies the trade-off between correctly identifying patients who will develop thrombosis and unnecessarily treating patients who will not.

### Feature importance and model interpretability

Feature importance was quantified using Random Forest Gini importance scores, which measure the total decrease in node impurity attributed to each feature across all trees. SHapley Additive exPlanations (SHAP) analysis was additionally performed on both the training and test sets to assess the directionality, magnitude, and consistency of individual feature contributions across datasets. SHAP rankings from training and test sets were compared to evaluate the stability of feature importance estimates (Additional file 4).

### Sensitivity analyses

Four sensitivity analyses were conducted to assess the robustness of the primary findings:

1. *Patient-clustered data splitting*: To address the statistical dependency arising from multiple PICC insertions per patient, a GroupShuffleSplit approach was used to ensure that all insertions from the same patient were allocated to either the training or the test set, preventing data leakage across patients. Additionally, a first-PICC-only analysis restricted the dataset to each patient’s first PICC insertion (n = 6,376).
2. *Multiple imputation*: To evaluate the impact of complete-case exclusion, multiple imputation using chained equations (MICE) was performed with the IterativeImputer (m = 5 imputed datasets). Each imputed dataset was independently analyzed, and results were pooled using Rubin’s rules to obtain combined point estimates and confidence intervals.
3. *Subgroup analysis without prior thrombosis history*: Given the dominant influence of prior thrombosis history on model predictions, a subgroup analysis was conducted on patients without any prior thrombosis history to evaluate model performance in a clinically less obvious population.
4. *Feature selection experiment*: To explore the feasibility of parsimonious models with fewer predictors, Random Forest models were trained using the top 5, 10, 15, 20, and 25 features ranked by Gini importance, and their performance was compared against the full 29-feature model (Additional file 5).

## Results

### Study population

**Fig. 1:**
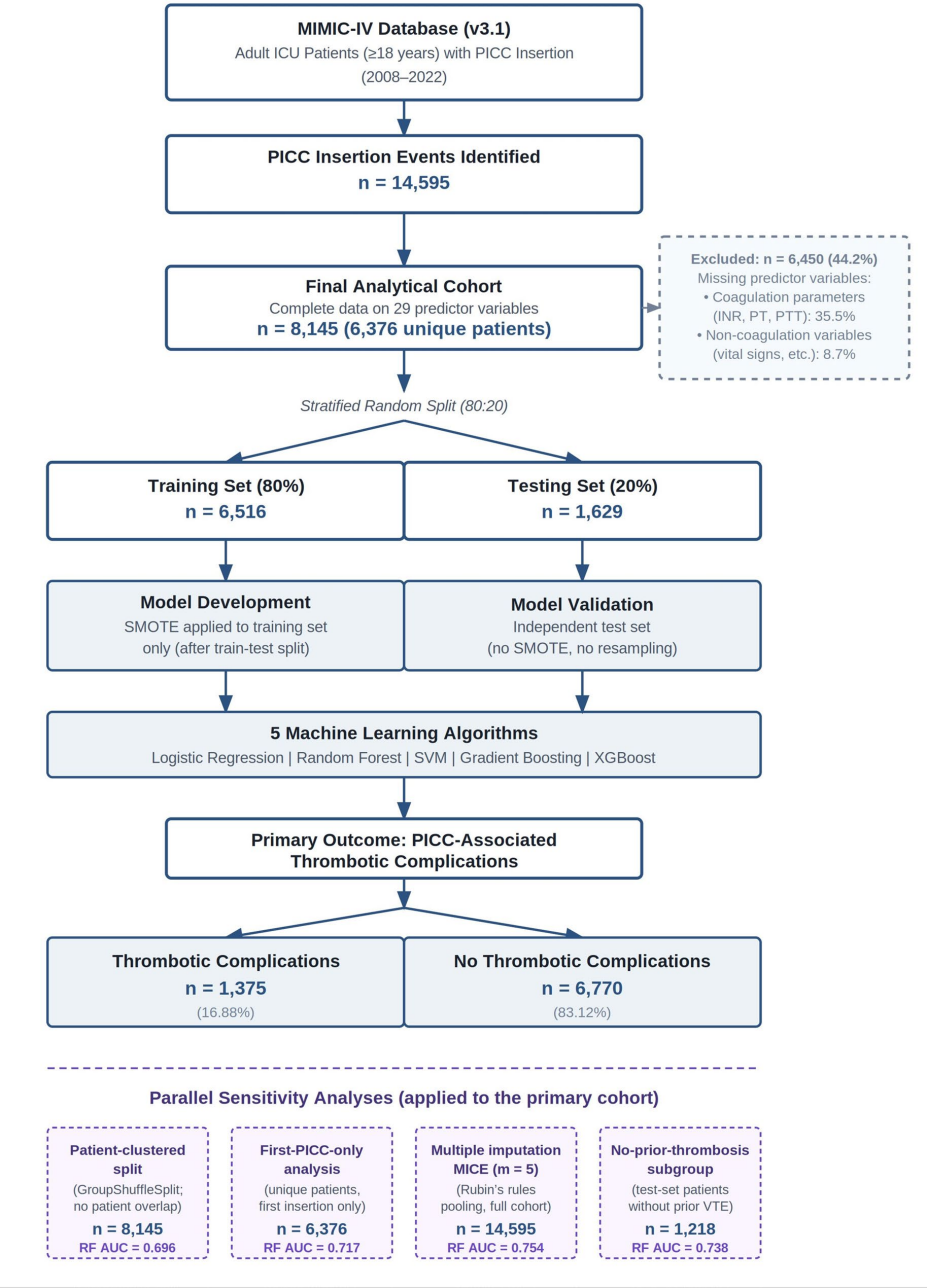
Study flow diagram. Patient selection process from the MIMIC-IV database (v3.1)

A total of 14,595 PICC insertion events were identified from the MIMIC-IV database (v3.1). After excluding 6,450 cases (44.2%) with missing predictor variables, the final analytical cohort comprised 8,145 PICC insertions from 6,376 unique critically ill patients admitted to intensive care units between 2008 and 2022 (Fig. 1). Thrombotic complications occurred in 1,375 cases (16.88%). Comparison of baseline characteristics between the training set (*n* = 6,516) and test set (*n* = 1,629) showed broad balance across the 29 predictor variables. Two variables (patient weight and cancer history) showed nominal *P* < 0.05 (*P* = 0.003 and *P* = 0.045, respectively); however, the corresponding effect sizes were small (mean weight difference 2.22 kg; cancer prevalence difference 2.6% points) and unlikely to materially affect model performance (Additional file 2). Table 1 presents baseline characteristics stratified by thrombotic complication status.

The cohort represented a critically ill population with substantial comorbidity burden (mean age 63.83 ± 15.70 years, 53.5% male). Patients who developed thrombosis did not differ significantly in age, gender, or body weight (all *P* > 0.05), but had longer intervals from hospital admission to PICC insertion (235.35 vs. 207.31 h, *P* = 0.0009). All three coagulation parameters were significantly higher in the thrombosis group: INR (1.54 vs. 1.49, *P* = 0.0364), PT (16.78 vs. 16.30 s, *P* = 0.0203), and PTT (42.39 vs. 38.58 s, *P* < 0.0001). The thrombosis group exhibited lower hemoglobin (9.37 vs. 9.58 g/dL, *P* = 0.0003) and higher platelet counts (235.98 vs. 226.29 × 10^9^/L, *P* = 0.0212). P-values were calculated using Welch’s t-test for continuous variables and the chi-square test for categorical variables. Given that some patients contributed multiple PICC insertions (8,145 insertions from 6,376 patients), these P-values should be interpreted with caution as the independence assumption may be partially violated.

Prior thrombosis history emerged as the most striking distinguishing characteristic, present in 51.9% of patients developing complications versus 18.4% without (*P* < 0.0001). Patients with prior thrombosis experienced more than three-fold higher thrombosis rates (36.4% vs. 10.7%, *P* < 0.0001). Active malignancy (32.1% vs. 28.0%, *P* = 0.0028) and CVD (62.1% vs. 55.3%, *P* < 0.0001) were also significantly more prevalent in the thrombosis group.

**Table 1:** Baseline characteristics of the study cohort

Group	Variable	Overall (*n* = 8,145)	Thrombosis (*n* = 1,375)	No thrombosis (*n* = 6,770)	*P*-value
*Demographics*	Age (years)	63.83 ± 15.70	64.05 ± 15.57	63.78 ± 15.73	0.5701
	Male gender	4356 (53.5%)	743 (54.0%)	3613 (53.4%)	0.6719
	Weight (kg)	84.79 ± 30.87	85.14 ± 27.93	84.71 ± 31.44	0.6398
*Clinical*	Time to PICC (hours)	212.05 ± 284.66	235.35 ± 311.43	207.31 ± 278.69	**0.0009**
*Coagulation*	INR	1.50 ± 0.66	1.54 ± 0.72	1.49 ± 0.65	**0.0364**
	PT (seconds)	16.38 ± 6.94	16.78 ± 7.59	16.30 ± 6.80	**0.0203**
	PTT (seconds)	39.23 ± 20.74	42.39 ± 23.93	38.58 ± 19.97	**< 0.0001**
*Hematologic*	Platelet (×10⁹/L)	227.93 ± 142.05	235.98 ± 143.23	226.29 ± 141.76	**0.0212**
	WBC (×10⁹/L)	12.42 ± 10.42	12.56 ± 12.66	12.39 ± 9.90	0.5842
	Hemoglobin (g/dL)	9.54 ± 1.89	9.37 ± 1.77	9.58 ± 1.91	**0.0003**
	RBC (×10¹²/L)	3.23 ± 0.68	3.19 ± 0.63	3.23 ± 0.69	**0.0444**
*Renal*	Creatinine (mg/dL)	1.43 ± 1.29	1.38 ± 1.24	1.44 ± 1.30	0.1429
	BUN (mg/dL)	32.86 ± 26.17	31.63 ± 24.25	33.11 ± 26.54	0.0563
*Vital signs*	Heart rate (bpm)	103.72 ± 12.79	104.00 ± 12.49	103.66 ± 12.85	0.3765
	MAP (mmHg)	79.59 ± 33.13	79.95 ± 17.54	79.51 ± 35.47	0.6552
	Temperature (°F)	98.35 ± 3.01	98.35 ± 0.94	98.35 ± 3.27	0.9952
	Resp. rate (/min)	20.73 ± 4.75	20.55 ± 4.61	20.76 ± 4.77	0.1396
	SpO₂ (%)	96.77 ± 2.58	96.82 ± 2.20	96.76 ± 2.65	0.4387
*Comorbidities*	Cancer	2339 (28.7%)	441 (32.1%)	1898 (28.0%)	**0.0028**
	Prior thrombosis	1961 (24.1%)	713 (51.9%)	1248 (18.4%)	**< 0.0001**
	CKD	2102 (25.8%)	361 (26.3%)	1741 (25.7%)	0.7025
	Diabetes mellitus	2509 (30.8%)	418 (30.4%)	2091 (30.9%)	0.7459
	CVD	4598 (56.5%)	854 (62.1%)	3744 (55.3%)	**< 0.0001**
*Medications*	Heparin	7238 (88.9%)	1217 (88.5%)	6021 (88.9%)	0.6801
	Warfarin	945 (11.6%)	228 (16.6%)	717 (10.6%)	**< 0.0001**
	DOAC	189 (2.3%)	69 (5.0%)	120 (1.8%)	**< 0.0001**
	Antiplatelet	2974 (36.5%)	502 (36.5%)	2472 (36.5%)	1.0000
	Mech. ventilation	2884 (35.4%)	451 (32.8%)	2433 (35.9%)	**0.0287**
	Vasopressor	1255 (15.4%)	206 (15.0%)	1049 (15.5%)	0.6604

Continuous variables: Mean ± SD. Categorical variables: n (%). Bold *P*-values: *P* < 0.05. *P*-values computed by Welch’s t-test (continuous) and chi-square test (categorical). INR, International Normalized Ratio; PT, Prothrombin Time; PTT, Partial Thromboplastin Time; WBC, White blood cell count; RBC, Red blood cell count; BUN, Blood urea nitrogen; MAP, Mean arterial pressure; SpO₂, Oxygen saturation; CKD, Chronic kidney disease; CVD, Cardiovascular disease; DOAC, Direct oral anticoagulant

### Model performance

**Fig. 2:**
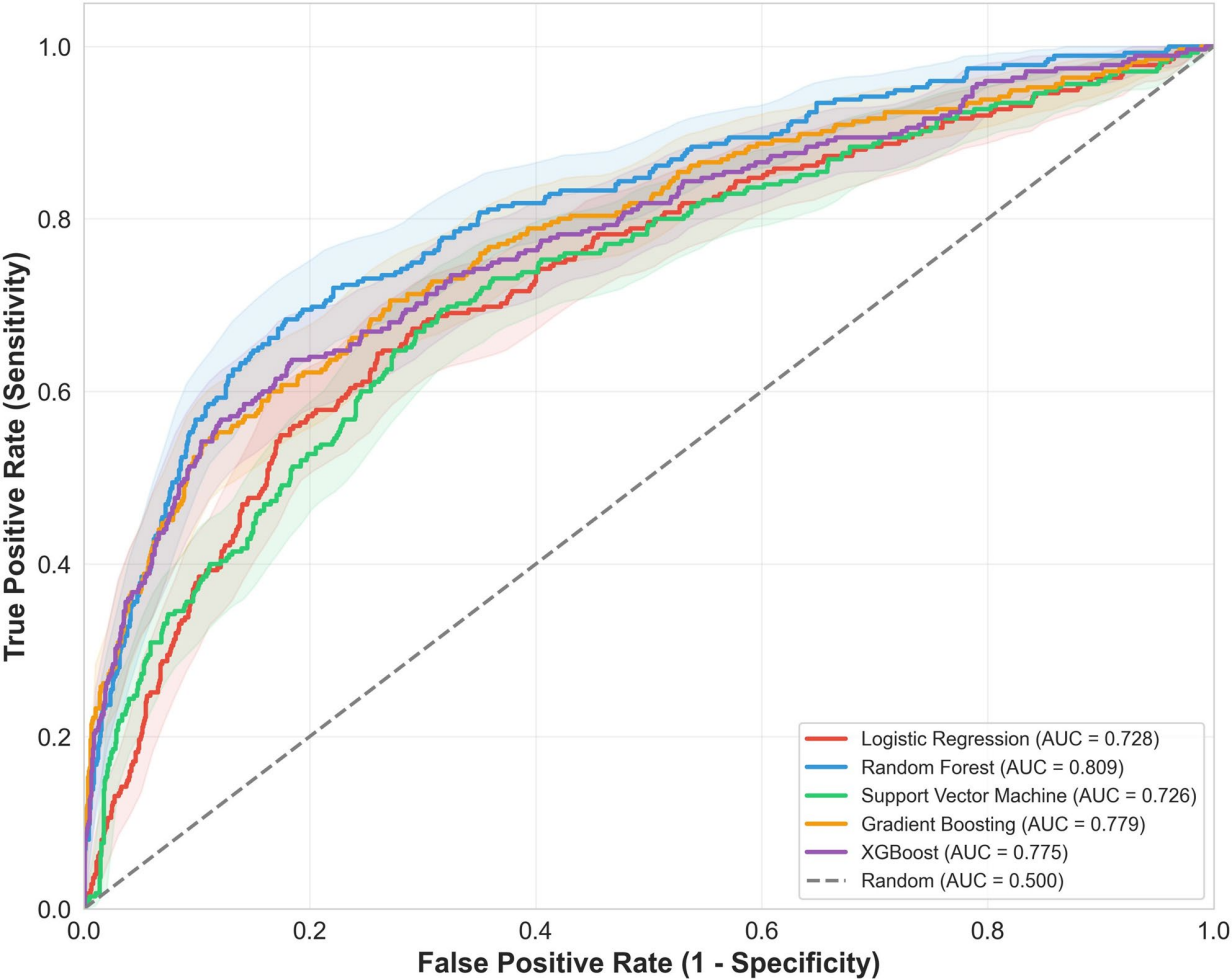
Receiver Operating Characteristic (ROC) curves with 95% bootstrap confidence bands comparing five machine learning models for predicting PICC-associated thrombotic complications. Shaded regions represent 95% CIs from 2,000 bootstrap resamples

Table 2 presents comprehensive performance metrics with bootstrap 95% confidence intervals (*n* = 2,000) for all five machine learning models evaluated on the independent test set (*n* = 1,629). The Random Forest model achieved the highest AUC-ROC of 0.809 (95% CI: 0.780–0.838; Fig. 2) and the best F1-score of 0.511 (95% CI: 0.458–0.564), exceeding the AUC-ROC threshold of 0.70 generally considered clinically useful [25]. The F1-score, defined as the harmonic mean of precision and sensitivity, provides a single metric that balances the trade-off between false positives and false negatives; a higher F1-score indicates a more balanced classifier performance in the presence of class imbalance.

**Fig. 3:**
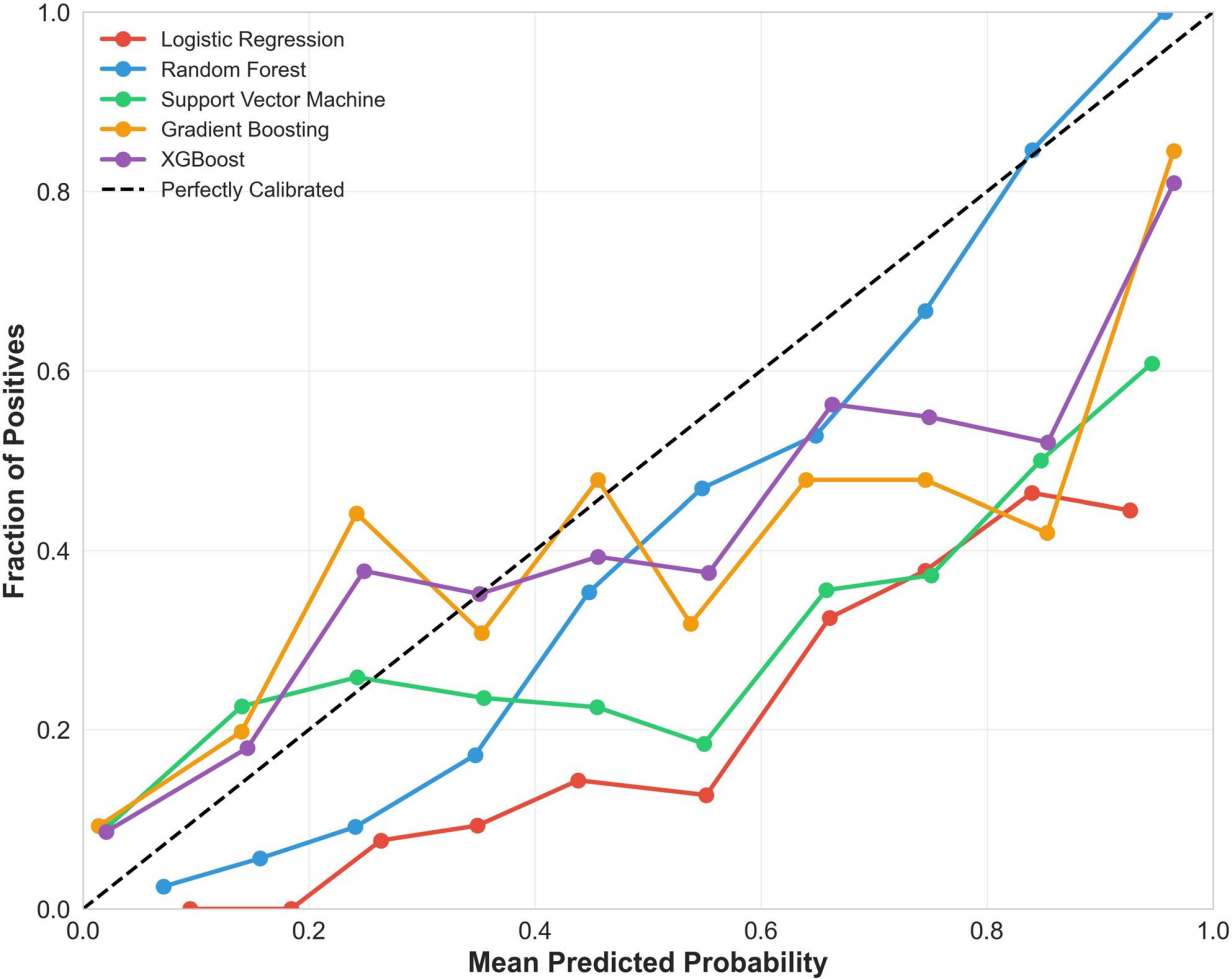
Calibration curves for five machine learning models

DeLong’s test confirmed that Random Forest significantly outperformed all other models: versus LR (ΔAUC = 0.081, z = 5.94, *P* < 0.0001), versus SVM (ΔAUC = 0.083, z = 5.65, *P* < 0.0001), versus GB (ΔAUC = 0.030, z = 3.12, *P* = 0.002), and versus XGB (ΔAUC = 0.035, z = 3.39, *P* < 0.001). Although GB and XGB achieved higher accuracy and specificity, these gains came at the cost of substantially reduced sensitivity. Calibration analysis showed that LR achieved the best calibration slope (0.949, closest to the ideal value of 1.0), while RF had a slope of 1.369, indicating mild underconfidence. SVM (slope 0.289), GB (0.389), and XGB (0.430) all showed substantially over-dispersed probability estimates, indicating that the magnitudes of their raw predicted probabilities should not be interpreted as well-calibrated event rates without further recalibration. Brier scores were lowest for the tree-based ensembles (XGB 0.117, GB 0.119, RF 0.121) and highest for LR (0.208) (Fig. 3; full metrics in Additional file 6).

Using the Youden index to determine optimal classification thresholds, the RF model achieved a threshold of 0.347 (Youden’s J = 0.505), yielding a sensitivity of 0.684 and specificity of 0.821 at this operating point—a substantially more balanced performance profile than the default 0.5 threshold (Table 2).

**Table 2:** Performance metrics with bootstrap 95% confidence intervals for five machine learning models on independent test set (*n* = 1,629)

Model	Accuracy (95% CI)	Precision (95% CI)	Sensitivity (95% CI)	Specificity (95% CI)	F1-Score (95% CI)	AUC-ROC (95% CI)	Brier	Cal. slope
LR	0.742 (0.720–0.762)	0.345 (0.302–0.389)	0.589 (0.527–0.646)	0.772 (0.749–0.795)	0.435 (0.388–0.478)	0.728 (0.692–0.761)	0.208	0.949
**RF**	**0.849 (0.832–0.866)**	**0.565 (0.498–0.631)**	**0.468 (0.409–0.528)**	**0.927 (0.912–0.941)**	**0.511 (0.458–0.564)**	**0.809 (0.780–0.838)**	**0.121**	**1.369**
SVM	0.790 (0.770–0.809)	0.386 (0.333–0.443)	0.414 (0.355–0.470)	0.866 (0.848–0.884)	0.399 (0.349–0.449)	0.726 (0.692–0.760)	0.146	0.289
GB	0.852 (0.835–0.868)	0.599 (0.528–0.674)	0.370 (0.314–0.426)	0.950 (0.938–0.961)	0.457 (0.399–0.513)	0.779 (0.745–0.812)	0.119	0.389
XGB	0.854 (0.836–0.870)	0.610 (0.536–0.686)	0.370 (0.314–0.427)	0.952 (0.940–0.964)	0.460 (0.403–0.518)	0.775 (0.740–0.808)	0.117	0.430

LR, Logistic Regression; RF, Random Forest (bold, best overall); SVM, Support Vector Machine; GB, Gradient Boosting; XGB, XGBoost. CIs computed by bootstrap resampling (*n* = 2,000). Brier score: lower is better (perfect = 0). Cal. Slope: calibration slope (ideal = 1.0). Calibration intercepts (ideal = 0): LR = − 1.612, RF = − 0.582, SVM = − 0.882, GB = − 0.382, XGB = − 0.445. Full calibration metrics (slope, intercept, Brier score) for all five models are provided as Additional file 6

### Decision curve analysis

**Fig. 4:**
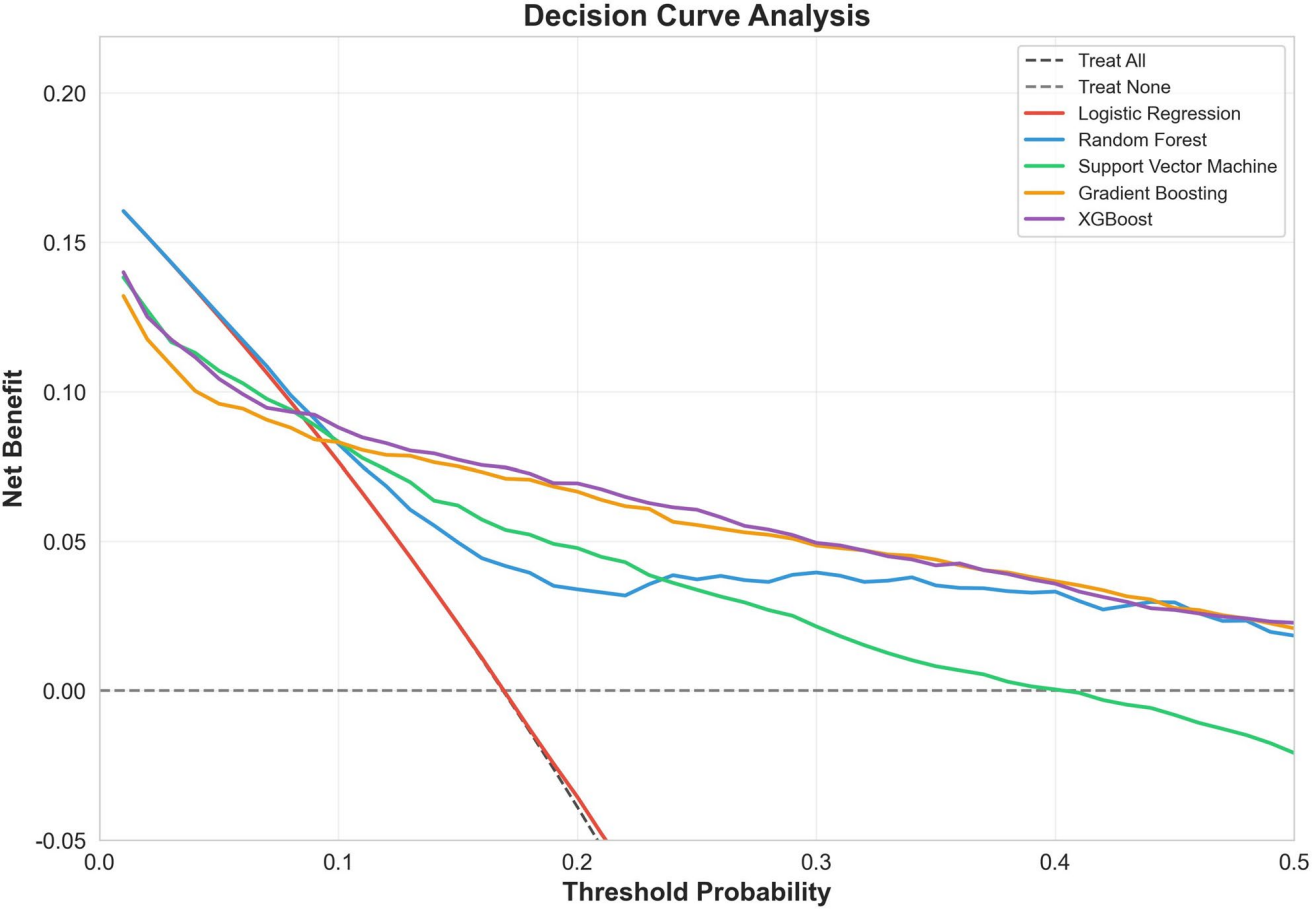
Decision curve analysis comparing five machine learning models. The x-axis represents threshold probability, and the y-axis represents net benefit. The dashed black line represents the “treat all” strategy, and the horizontal gray line represents the “treat none” strategy

Decision curve analysis (Fig. 4) showed that the three tree-based ensembles (Random Forest, Gradient Boosting, XGBoost) provided net clinical benefit substantially exceeding both the “treat all” and “treat none” default strategies across the clinically relevant threshold range of approximately 1–50%. At the lowest thresholds (~ 1–8%), Random Forest achieved the highest net benefit. At intermediate-to-higher thresholds (~ 10–40%), Gradient Boosting and XGBoost provided the highest net benefit, consistent with their high-specificity operating characteristics. Logistic Regression provided competitive net benefit at very low thresholds (~ 1–8%) but fell below the “treat none” line at thresholds beyond approximately 18%, reflecting its lower specificity at those operating points. Support Vector Machine provided positive net benefit up to approximately 40% but fell below “treat none” thereafter. Overall, the three tree-based ensembles supported clinical utility for risk stratification across the broadest threshold range.

### Feature importance

**Fig. 5:**
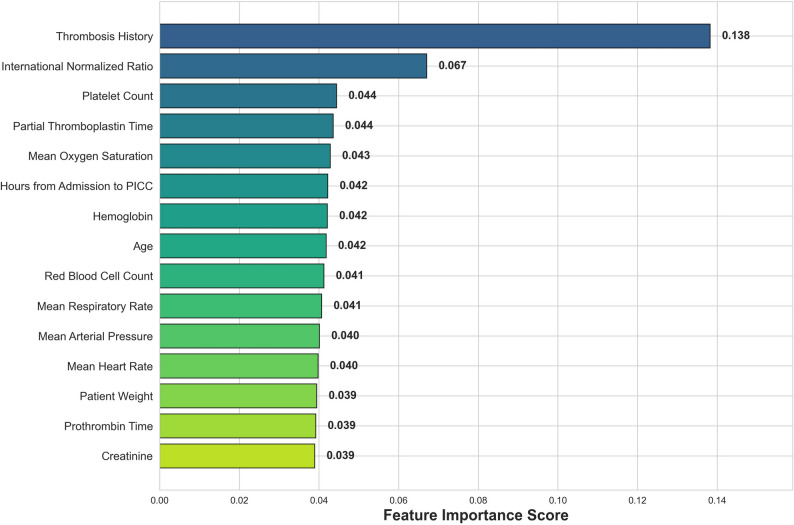
Top 15 most important features for predicting PICC-associated thrombotic complications

Feature importance analysis using the RF model revealed a clear hierarchy of predictive contribution among the 29 clinical features (Fig. 5). Prior thrombosis history emerged as the single most important predictor (importance score 0.138), substantially exceeding all other features. INR (0.067) ranked second, followed by platelet count (0.044), PTT (0.044), mean oxygen saturation (0.043), time from hospital admission to PICC insertion (0.042), hemoglobin (0.042), age (0.042), RBC count (0.041), respiratory rate (0.041), MAP (0.040), mean heart rate (0.040), patient weight (0.039), PT (0.039), and creatinine (0.039). The top 15 features represented diverse clinical domains including patient history, coagulation parameters, hematologic indices, vital signs, temporal factors, and demographics.

### Feature selection experiment

**Fig. 6:**
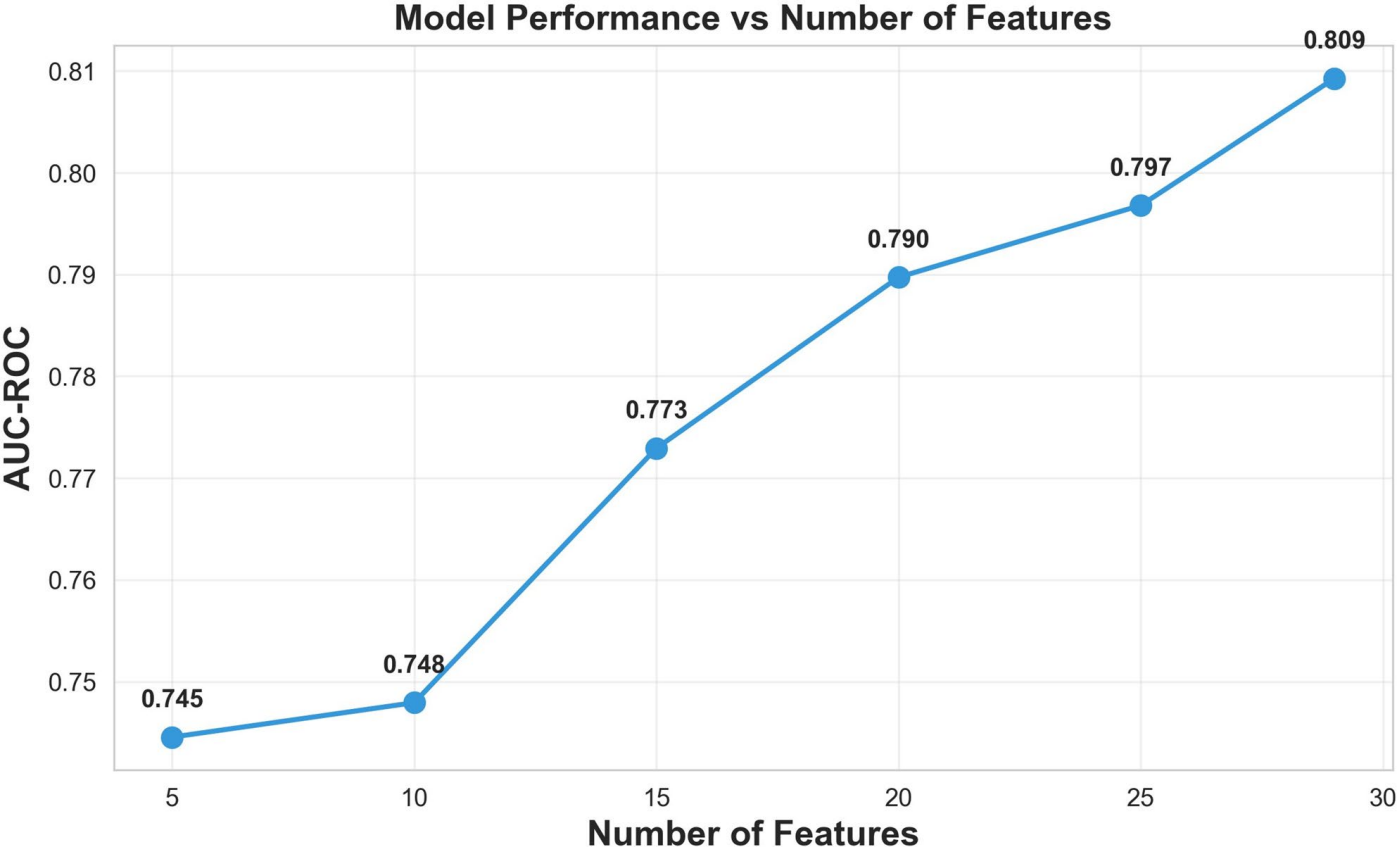
Model performance (AUC-ROC) as a function of the number of features included, ranked by Gini importance

To evaluate the feasibility of more parsimonious models, RF models were trained using subsets of the top-ranked features (Fig. 6; Additional file 5). A model using only the top 15 features achieved an AUC-ROC of 0.773, retaining 95.5% of the full model’s discriminative ability (0.809). Even a minimal model with 5 features achieved an AUC-ROC of 0.745. The performance curve showed diminishing marginal returns beyond approximately 20 features (AUC = 0.790), suggesting that a parsimonious model with 15–20 features could provide clinically acceptable performance with reduced data requirements.

### Model interpretability

**Fig. 7:**
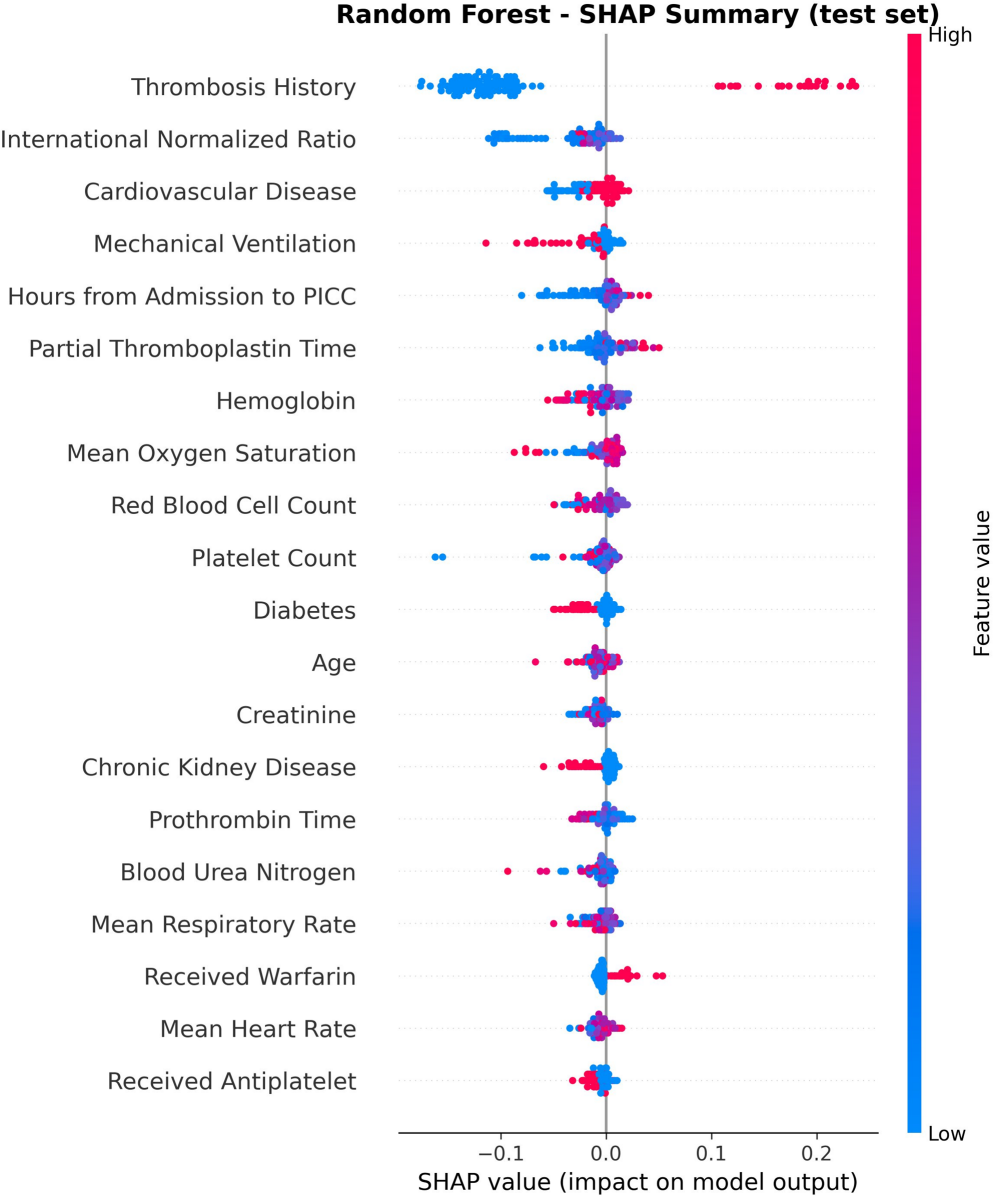
SHAP summary plot for the Random Forest model on the test set. Each dot represents one patient; the horizontal position indicates the SHAP value (impact on model output), and the colour indicates the feature value (red = high, blue = low)

SHAP analysis was performed on both the training and test sets to assess the stability of feature importance estimates across datasets (Fig. 7; Additional file 4). On the test set, the SHAP summary plot confirmed prior thrombosis history as the most influential predictor, with high feature values (presence of thrombosis history) strongly pushing predictions toward the thrombotic complication class (mean |SHAP| = 0.131). INR ranked second in both SHAP and Gini importance analyses. Feature importance rankings were highly consistent between training and test sets, with Spearman rank correlation exceeding 0.90 for the RF model, supporting the stability and generalizability of the identified predictive patterns.

Notable ranking differences between Gini importance and SHAP emerged: CVD and mechanical ventilation ranked third and fourth by SHAP but were absent from the Gini-based top 15, likely because binary variables generate fewer tree splits despite exerting substantial per-sample effects. Conversely, platelet count ranked third by Gini importance but tenth by SHAP, suggesting frequent use as a splitting variable with relatively modest individual-level impact. The SHAP analysis further revealed bidirectional effects for continuous variables: higher INR and PTT values were associated with increased thrombosis risk, while lower hemoglobin and SpO_2_ values contributed to elevated risk predictions.

### Sensitivity analyses

Four sensitivity analyses were conducted to assess the robustness of the primary findings (Table 3).

#### *Patient-clustered splitting*

When data were split by patient identity to ensure zero patient overlap between training and test sets, the RF model achieved an AUC-ROC of 0.696, representing a 14% relative decrease from the primary analysis (0.809). This decrease confirms that some performance in the primary analysis was attributable to within-patient correlation across multiple PICC insertions, consistent with concerns regarding statistical dependency. When restricted to each patient’s first PICC insertion only (n = 6,376), the model achieved an AUC-ROC of 0.717, demonstrating acceptable discriminative ability independent of repeated measures.

#### *Multiple imputation*

Using MICE (m = 5, Rubin’s rules), the RF model achieved a pooled AUC-ROC of 0.754 (95% CI: 0.747–0.760) on the full dataset (n = 14,595). The modest decrease from the complete-case AUC (0.809) likely reflects both the dilution of predictive signal by imputed values and the inclusion of patients with systematically different clinical profiles. Importantly, the direction and relative ranking of feature importance were preserved across imputed datasets.

#### *Subgroup without prior thrombosis history*

Among test set patients without prior thrombosis history (n = 1,218, event rate 10.10%), the RF model maintained an AUC-ROC of 0.738 (Additional file 7). Although lower than the full-cohort AUC, this demonstrates that the model retains meaningful discriminative ability even when the dominant predictor is absent, relying on coagulation parameters, hematologic indices, and other clinical features.

**Table 3:** Sensitivity analysis results for the Random Forest model

Analysis	*N*	Event rate	AUC-ROC	F1-score
Primary analysis (complete case)	8,145	16.88%	0.809	0.511
Patient-clustered split	8,145	16.88%	0.696	0.315
First PICC only	6,376	15.98%	0.717	—
Multiple imputation (pooled, m = 5)	14,595	16.22%	0.754	0.451
Subgroup: no prior thrombosis	1,218	10.10%	0.738	0.387

## Discussion

This study evaluates machine learning models for predicting PICC-associated thrombotic complications in critically ill patients. The Random Forest model achieved an AUC-ROC of 0.809 (95% CI: 0.780–0.838) in the primary complete-case analysis, with a calibration slope of 1.369 indicating mild underconfidence; a patient-clustered sensitivity analysis (AUC = 0.696) further indicates that part of the primary-analysis performance reflects within-patient correlation in repeated insertions [25]. DeLong’s test confirmed statistically significant superiority over all alternative algorithms (all *P* < 0.01), and DCA demonstrated net clinical benefit across threshold probabilities of approximately 1–50% for the three tree-based ensembles.

Prior thrombosis history emerged as the dominant predictor (feature importance 13.8%), with patients having prior thrombosis experiencing more than three-fold higher thrombosis rates (36.4% vs. 10.7%, *P* < 0.0001). This finding has clear clinical implications: comprehensive medical history documentation during admission assessments is a critical patient safety intervention that directly determines risk prediction accuracy and preventive care effectiveness. Importantly, the subgroup analysis demonstrated that the model retained meaningful discriminative ability (AUC = 0.738) even among patients without prior thrombosis history, relying on coagulation parameters, hematologic indices, and temporal factors to stratify risk in this clinically less obvious population. The model’s discriminative profile may support clinical decision support: at the Youden-optimal threshold of 0.347, the RF model achieves sensitivity 0.684 and specificity 0.821, identifying a high-risk subgroup for targeted prophylaxis while accepting that roughly one in three thrombotic events would still be missed at this operating point. The choice of operating threshold should ultimately be calibrated to the clinical cost trade-off between under-prophylaxis and over-anticoagulation.

The feature selection experiment demonstrated that parsimonious models with as few as 15 features retained 95.5% of the full model’s discriminative ability (AUC 0.773 vs. 0.809). This finding is clinically significant because a reduced-feature model would lower the data completeness barrier that led to the exclusion of 44.2% of the original cohort. Future clinical implementations could prioritize a core set of 15–20 highly predictive features, improving model applicability while maintaining acceptable performance. Transitioning these findings into a practical clinical risk score would involve several steps: selecting the optimal parsimonious feature subset, calibrating probability estimates to the target population, and integrating the model into EHR systems as an automated alert triggered at the time of PICC order placement. All 29 predictor variables used in this study are routinely collected during ICU care and available through standard EHR interfaces, ensuring feasibility of real-time implementation. Regarding the 24-hour laboratory extraction window, the tool is designed to use the most recent available values at the time of PICC order; in practice, ICU patients typically have laboratory panels updated at least daily, making this requirement readily achievable. In rare cases where a value is more than 24 h old, the system could flag the prediction as provisional and recommend updated testing.

The elevated coagulation parameters (INR, PT, PTT) in the thrombosis group present a clinically important paradox. These counterintuitive findings likely reflect complex coagulopathies such as disseminated intravascular coagulation (DIC) or severe hepatic dysfunction, where prolonged coagulation times coexist with increased thrombotic risk. SHAP analysis provided direct evidence for this relationship, demonstrating that higher INR and PTT values shifted model predictions toward increased thrombosis risk (Fig. 7), confirming that the model appropriately captured this non-linear, paradoxical association. Capturing such non-linear, paradoxical associations may be a strength of flexible learners; whether this translates into incremental clinical value compared with established scoring tools such as the Caprini score requires direct head-to-head comparison, which we did not perform. Similarly, lower hemoglobin and higher platelet counts in the thrombosis group are consistent with known pathophysiologic mechanisms involving blood viscosity alterations and platelet activation in thrombogenesis, and SHAP analysis confirmed the directionality of these effects at the individual patient level.

Recent comparative analyses have demonstrated that PICC-specific risk assessment models achieve superior accuracy compared to generic thrombosis risk models [26], supporting the development of targeted prediction tools as presented in this study. The systematic comparison of five diverse machine learning algorithms demonstrated consistency across different modeling approaches, with all achieving clinically meaningful discrimination (AUC > 0.72), suggesting robust rather than algorithm-specific findings. LightGBM was not included as it shares the same gradient boosting framework as XGBoost and GB, and preliminary comparisons showed no meaningful performance differences. Deep learning was considered less appropriate given the tabular data structure and modest sample size, where tree-based ensembles have been shown to outperform neural networks [21].

The complementary use of Gini importance and SHAP analysis performed on both training and test sets provided a more nuanced and robust understanding of model behaviour than either method alone. Feature importance rankings were highly consistent between the two datasets, supporting the generalizability of the identified predictive patterns. While Gini importance quantified overall predictive contribution, SHAP analysis revealed clinically meaningful effect directions and uncovered features whose individual-level impact was underestimated by tree-based splitting metrics. Notably, CVD ranked third by SHAP despite its absence from the Gini top 15, highlighting that binary comorbidity variables can exert substantial effects on individual predictions even when infrequently selected as splitting nodes. This dual-method, dual-dataset approach enhances clinical trust by demonstrating not only which features matter most, but how they influence predictions for individual patients.

### Strengths and limitations

This study has several methodologic strengths. The large sample size exceeding 8,000 PICC insertions provides robust statistical power. The systematic evaluation of 29 variables across seven clinically relevant domains offers comprehensive risk assessment. The rigorous comparison of five diverse algorithms with grid search cross-validated hyperparameter optimization demonstrates consistency across modeling approaches. The use of bootstrap confidence intervals, DeLong’s test, DCA, and multiple sensitivity analyses provides a thorough evaluation of model performance and robustness. The quantitative feature importance analysis using both Gini importance and SHAP values across both training and test sets provides clinical interpretability, transparency, and demonstrated stability.

However, several limitations should be acknowledged. First, the retrospective single-center design and the 14-year cohort span (2008–2022) limit generalizability: we did not perform formal temporal validation, and clinical practice in PICC placement, anticoagulation, and outcome documentation may have shifted over this interval. External validation in multi-center and more recent datasets is therefore needed before clinical implementation. Second, outcome ascertainment through ICD diagnosis codes likely underestimates true thrombosis incidence by missing asymptomatic thrombi. Administrative codes are known to have limited sensitivity for identifying DVT and catheter-related thrombosis compared to systematic ultrasound surveillance. The reported incidence of 16.88% may therefore underestimate asymptomatic but clinically relevant thrombi. This misclassification bias, specifically false negatives in the non-thrombosis group, may attenuate the model’s sensitivity and positive predictive value. While the model captures clinically significant events requiring intervention, future studies using systematic ultrasound surveillance as the reference standard would better characterize subclinical thrombotic events.

Third, the complete-case analysis excluded 44.2% of PICC insertions, primarily due to missing coagulation parameters (35.5%), which is common in routine clinical practice where not all ICU patients receive comprehensive coagulation testing. This introduces potential selection bias, as patients who receive comprehensive coagulation panels may be systematically different from those who do not—potentially representing a sicker or more coagulopathic subpopulation. The multiple imputation sensitivity analysis (pooled AUC = 0.754) confirmed that the model’s conclusions remained directionally consistent on the full dataset, though with expectedly attenuated discrimination. Nevertheless, the model may be most applicable to patients for whom complete coagulation panels are available.

Fourth, the analysis of 8,145 PICC insertions from 6,376 patients means that some patients contributed multiple observations. This violates the independence assumption of standard machine learning algorithms. The patient-clustered splitting sensitivity analysis (AUC = 0.696) and first-PICC-only analysis (AUC = 0.717) demonstrated that within-patient correlation contributed to the primary analysis performance. While the model retained acceptable discriminative ability in these analyses, future implementations should consider patient-level grouping during model development and validation to prevent optimistic performance estimates.

Fifth, the model lacks critical catheter-specific variables including catheter gauge, number of lumens, and insertion site. Because larger bore catheters mechanically obstruct venous flow, these variables represent primary physical drivers of catheter-related thrombosis pathology. Their absence means this model functions as a “patient-factor” risk tool rather than a comprehensive “procedure-factor” risk tool. These variables were unavailable in MIMIC-IV but should be incorporated in future studies where such data are available.

Sixth, the model is not yet deployment-ready in two related senses: the study did not include head-to-head comparison with existing clinical risk scores (e.g., the Caprini score) applied to the same cohort, so the incremental value of machine learning over conventional tools is not directly quantified; and the calibration analysis revealed mild under-confidence of the RF model (slope 1.369), suggesting that pre-deployment recalibration (for example, Platt scaling or isotonic regression on a local cohort) would be advisable before predicted probabilities are used in clinical decision-making.

## Conclusions

In this MIMIC-IV cohort, the Random Forest model showed acceptable discrimination for PICC-associated thrombotic complications using routinely collected clinical data, with the caveat that performance was attenuated under patient-clustered splitting. The RF model achieved an AUC-ROC of 0.809 (95% CI: 0.780–0.838), significantly outperforming all alternative algorithms, with DCA confirming net clinical benefit across a clinically relevant range of threshold probabilities. Prior thrombosis history emerged as the dominant predictor, underscoring the importance of comprehensive medical history documentation. Feature selection experiments demonstrated that parsimonious models with 15–20 features retained over 95% of discriminative performance, supporting the feasibility of streamlined clinical implementation. Sensitivity analyses confirmed directional robustness of findings across patient-clustered splitting, multiple imputation, and subgroup scenarios. External validation in multi-center settings and prospective implementation studies are warranted to translate these findings into clinical practice.

## Supplementary Information

Below is the link to the electronic supplementary material.

Supplementary Material 1: Additional file 1 — TRIPOD + AI checklist. File format: .pdf Description: Completed TRIPOD + AI checklist indicating the page number for each reported item.

Supplementary Material 2: Additional file 2 — Comparison of baseline characteristics between training and test sets. File format: .csv Description: Table comparing all 29 predictor variables and the outcome variable between the training set (n = 6,516) and test set (n = 1,629), with P-values from Welch’s t-test (continuous) and chi-square test (categorical). Two variables (patient weight, *P* = 0.003; cancer history, *P* = 0.045) showed nominal *P* < 0.05 with small effect sizes; all other 27 variables showed no statistically significant imbalance.

Supplementary Material 3: Additional file 3 — Grid search hyperparameter spaces and optimal parameters. File format: .json Description: Grid search spaces explored for each model and the optimal hyperparameters identified through five-fold stratified cross-validation.

Supplementary Material 4: Additional file 4 — SHAP feature importance rankings: training set versus test set comparison. File format: .csv Description: SHAP-based feature importance rankings for four machine learning models (LR, RF, GB, XGB) computed separately on training and test sets, enabling comparison of feature importance stability across datasets.

Supplementary Material 5: Additional file 5 — Feature selection experiment results. File format: .csv Description: Performance metrics (AUC-ROC, accuracy, precision, sensitivity, specificity, F1-score) for Random Forest models trained with the top 5, 10, 15, 20, and 25 features ranked by Gini importance, compared against the full 29-feature model.

Supplementary Material 6: Additional file 6 — Calibration metrics for all five machine learning models. File format: .csv Description: Calibration slope, calibration intercept, and Brier score for Logistic Regression, Random Forest, Support Vector Machine, Gradient Boosting, and XGBoost, computed on the independent test set (n = 1,629).

Supplementary Material 7: Additional file 7 — Subgroup analysis in patients without prior thrombosis history. File format: .csv Description: Performance metrics for all five machine learning models evaluated on test set patients without prior thrombosis history (n = 1,218, event rate 10.10%).

## Data Availability

The dataset supporting the conclusions of this article is available in the MIMIC-IV repository (version 3.1), https://physionet.org/content/mimiciv/3.1/, after completion of required training and a data use agreement. The analysis code is available from the corresponding author upon reasonable request.

## Abbreviations

AUC-ROC: Area Under the Receiver Operating Characteristic Curve
BUN: Blood Urea Nitrogen
Cal.: Calibration
CI: Confidence Interval
CKD: Chronic Kidney Disease
CVD: Cardiovascular Disease
DCA: Decision Curve Analysis
DIC: Disseminated Intravascular Coagulation
DOAC: Direct Oral Anticoagulant
DVT: Deep Vein Thrombosis
EHR: Electronic Health Record
GB: Gradient Boosting
ICD: International Classification of Diseases
ICU: Intensive Care Unit
INR: International Normalized Ratio
LR: Logistic Regression
MAP: Mean Arterial Pressure
MICE: Multiple Imputation by Chained Equations
MIMIC-IV: Medical Information Mart for Intensive Care IV
PE: Pulmonary Embolism
PICC: Peripherally Inserted Central Catheter
PT: Prothrombin Time
PTT: Partial Thromboplastin Time
RBC: Red Blood Cell
RBF: Radial Basis Function
RF: Random Forest
SHAP: SHapley Additive exPlanations
SMOTE: Synthetic Minority Over-sampling Technique
SpO_2_: Oxygen Saturation
SVM: Support Vector Machine
TRIPOD + AI: Transparent Reporting of a multivariable prediction model for Individual Prognosis Or Diagnosis plus Artificial Intelligence
WBC: White Blood Cell
XGB: XGBoost
