# Supplementary material for: Machine learning prediction of PICC-associated thrombotic complications in critically ill patients
Source: BMC Med Inform Decis Mak. 2026 May 30;26:287. doi: 10.1186/s12911-026-03607-w (PMC13439908; doi:10.1186/s12911-026-03607-w)
Supplement: Supplementary file 1 [file 12911_2026_3607_MOESM1_ESM.pdf]

Additional file 1

TRIPOD+AI Checklist

Machine learning prediction of PICC-associated thrombotic complications in critically ill patients

Reference: Collins GS, Moons KGM, Dhiman P, Riley RD, Beam AL, Van Calster B, et al. TRIPOD+AI statement: updated guidance for reporting clinical prediction models that use regression or machine learning methods. *BMJ*. 2024;385:e078378. doi:10.1136/bmj-2023-078378.

Type column key: D = items relevant to model development; E = items relevant to model evaluation; D;E = items applicable to both. This study reports model development with internal validation, so all D and D;E items are required. E-only items (12f, 12g, 20c, 24) are addressed where relevant or marked as not applicable.

Section / Topic	Item	Type	Checklist item	Reported in manuscript
**TITLE**				
Title	1	D;E	Identify the study as developing or evaluating the performance of a multivariable prediction model, the target population, and the outcome to be predicted.	Title (Page 1) — explicitly identifies "machine learning prediction" of "PICC-associated thrombotic complications" in "critically ill patients".
**ABSTRACT**				
Abstract	2	D;E	See TRIPOD+AI for Abstracts checklist.	Abstract (Page 1) — structured Background / Methods / Results / Conclusions; addresses each item required by TRIPOD+AI for Abstracts.
**INTRODUCTION**				
Background	3a	D;E	Explain the healthcare context (including whether diagnostic or prognostic) and rationale for developing or evaluating the prediction model, including references to existing models.	Background, paragraphs 1–3 — describes ICU PICC use, thrombosis incidence, pathophysiology, and limitations of existing tools (Caprini score) [refs 1–11,17,19].
Background	3b	D;E	Describe the target population and the intended purpose of the prediction model in the context of the care pathway, including its intended users (e.g., healthcare professionals, patients, public).	Background, paragraph 4 — target population: critically ill patients receiving PICCs in ICU; intended use: clinical decision support for thrombosis risk stratification by ICU clinicians.
Background	3c	D;E	Describe any known health inequalities between sociodemographic groups.	Not specifically addressed for the study setting. The cohort was drawn from a single tertiary care center (Beth Israel Deaconess Medical Center, Boston, USA) via the MIMIC-IV database; sociodemographic inequalities in PICC-associated thrombosis are not well established and were not a study objective. This is acknowledged as a limitation requiring multi-center external validation in diverse populations.
Objectives	4	D;E	Specify the study objectives, including whether the study describes the development or validation of a prediction model (or both).	Background, final paragraph — explicitly states the study aims to "develop and validate comprehensive machine learning models" (development with internal validation via independent test set).
**METHODS**				
Data	5a	D;E	Describe the sources of data separately for the development	Methods → Study design and data

Section / Topic	Item	Type	Checklist item	Reported in manuscript
			and evaluation datasets (e.g., randomised trial, cohort, routine care or registry data), the rationale for using these data, and representativeness of the data.	source — retrospective cohort from MIMIC-IV v3.1 [ref 20]; both development (training) and evaluation (test) datasets derived from the same source via stratified random partitioning. Rationale for MIMIC-IV: large publicly available ICU dataset suitable for ML model development.
Data	5b	D;E	Specify the dates of the collected participant data, including start and end of participant accrual; and, if applicable, end of follow-up.	Methods → Study design and data source — admissions between 2008 and 2022. Outcome ascertained during same hospitalization (no longitudinal follow-up beyond discharge).
Participants	6a	D;E	Specify key elements of the study setting (e.g., primary care, secondary care, general population) including the number and location of centres.	Methods → Study design and data source — single-center: Intensive Care Units of Beth Israel Deaconess Medical Center, Boston, MA, USA (tertiary academic medical center).
Participants	6b	D;E	Describe the eligibility criteria for study participants.	Methods → Study design and data source — adult patients (≥18 years) admitted to ICU receiving PICC insertion. Exclusion: missing predictor variables (n = 6,450, 44.2%). See Figure 1 (study flow diagram).
Participants	6c	D;E	Give details of any treatments received, and how they were handled during model development or evaluation, if relevant.	Methods → Predictor variables — Treatment variables explicitly included as model predictors: heparin, warfarin, DOACs, antiplatelet therapy, mechanical ventilation, vasopressors. Documented within 48 hours before PICC insertion (i.e., before outcome).
Data preparation	7	D;E	Describe any data pre-processing and quality checking, including whether this was similar across relevant sociodemographic groups.	Methods → Statistical analysis — z-score standardization for continuous variables; numeric encoding for categorical variables. Pre-processing applied uniformly across the entire cohort. Code available upon request.
Outcome	8a	D;E	Clearly define the outcome that is being predicted and the time horizon, including how and when assessed, the rationale for choosing this outcome, and whether the method of outcome assessment is consistent across sociodemographic groups.	Methods → Outcome definition — binary outcome (1 = thrombosis, 0 = no thrombosis); ascertained via ICD-9/ICD-10 codes during the same hospitalization as PICC insertion. Includes upper extremity venous thrombosis, DVT, superficial thrombophlebitis, catheter-related thrombosis, and PE. Time horizon: same hospital admission. Limitations of ICD-based ascertainment discussed in Strengths and limitations.
Outcome	8b	D;E	If outcome assessment requires subjective interpretation, describe the qualifications and demographic characteristics of the outcome assessors.	Not applicable — outcome was ascertained via standardized ICD-9/ICD-10 administrative codes (objective) rather than subjective adjudication.
Outcome	8c	D;E	Report any actions to blind assessment of the outcome to be predicted.	Not applicable — retrospective ICD-coded outcome ascertainment is independent of the modelling process; outcome status was not visible during predictor extraction.

Section / Topic	Item	Type	Checklist item	Reported in manuscript
Predictors	9a	D	Describe the choice of initial predictors (e.g., literature, previous models, all available predictors) and any pre-selection of predictors before model building.	Methods → Predictor variables — 29 predictors selected a priori based on established thrombosis risk factors from the literature [refs 9, 10] across seven clinical domains. No data-driven pre-selection before model building.
Predictors	9b	D;E	Clearly define all predictors, including how and when they were measured (and any actions to blind assessment of predictors for the outcome and other predictors).	Methods → Predictor variables — all 29 predictors fully defined with units and measurement windows: laboratory values within 24 hours pre-insertion; vital signs averaged over 24-hour pre-insertion period; medications within 48 hours before insertion. All predictor measurements precede the outcome event.
Predictors	9c	D;E	If predictor measurement requires subjective interpretation, describe the qualifications and demographic characteristics of the predictor assessors.	Not applicable — all 29 predictors are objective measurements (laboratory values, vital signs, demographic data, ICD-coded comorbidities, medication administration records).
Sample size	10	D;E	Explain how the study size was arrived at (separately for development and evaluation), and justify that the study size was sufficient to answer the research question. Include details of any sample size calculation.	Methods → Study design and data source / Results → Study population — all eligible records used (n = 8,145 PICC insertions; 1,375 events). Development: n = 6,516 (training, 80%). Evaluation: n = 1,629 (test, 20%). With 1,100 events in training (events per variable ≈ 38), the sample size considerably exceeds the minimum recommended for reliable model development.
Missing data	11	D;E	Describe how missing data were handled. Provide reasons for omitting any data.	Methods → Statistical analysis — primary analysis: complete-case (excluded 6,450 records with any missing predictor, primarily missing coagulation values [35.5%]). Sensitivity analysis: multiple imputation by chained equations (MICE; m = 5; sample_posterior = TRUE) with results pooled via Rubin's rules (Methods → Sensitivity analyses; Results → Multiple imputation; Additional file 4 derived statistics). No imputation across train/test boundary.
Analytical methods	12a	D	Describe how the data were used (e.g., for development and evaluation of model performance) in the analysis, including whether the data were partitioned, considering any sample size requirements.	Methods → Statistical analysis — stratified random 80/20 partitioning into training (n = 6,516) and independent test set (n = 1,629). Hyperparameter tuning conducted via 5-fold stratified cross-validation within the training set only. Test set used exclusively for final performance evaluation. No data leakage.
Analytical methods	12b	D	Depending on the type of model, describe how predictors were handled in the analyses (functional form, rescaling, transformation, or any standardisation).	Methods → Statistical analysis — continuous predictors z-score standardized; categorical variables encoded numerically (binary coding for sex, comorbidities, treatments). No transformations of functional form (raw predictors retained for tree-based models which are scale-invariant; standardization primarily for LR and

Section / Topic	Item	Type	Checklist item	Reported in manuscript
				SVM).
Analytical methods	12c	D	Specify the type of model, rationale, all model-building steps, including any hyperparameter tuning, and method for internal validation.	Methods → Statistical analysis and machine learning — five algorithms with explicit rationale for selection: Logistic Regression (linear baseline); Random Forest, Gradient Boosting, XGBoost (tree-based ensembles, suited for tabular data); SVM-RBF (kernel-based). LightGBM and deep learning excluded with justification [ref 23]. Hyperparameter tuning: exhaustive grid search with 5-fold stratified CV on AUC-ROC. Grid spaces and optimal parameters in Additional file 3.
Analytical methods	12d	D;E	Describe if and how any heterogeneity in estimates of model parameter values and model performance was handled and quantified across clusters (e.g., hospitals, countries).	Methods → Sensitivity analyses (Patient-clustered data splitting) — within-patient clustering addressed via GroupShuffleSplit ensuring all PICC insertions from one patient remained in either training or test set. Additional first-PICC-only analysis (n = 6,376 unique patients). Single-center study, so between-center heterogeneity not assessable; stated as limitation.
Analytical methods	12e	D;E	Specify all measures and plots used (and their rationale) to evaluate model performance (e.g., discrimination, calibration, clinical utility) and, if relevant, to compare multiple models.	Methods → Model evaluation / Decision curve analysis — Discrimination: AUC-ROC (primary), accuracy, precision, sensitivity, specificity, F1. Calibration: calibration plot, calibration slope, calibration intercept, Brier score (Figure 3). Clinical utility: decision curve analysis (Figure 4). Comparison between models: DeLong's test for paired AUC-ROC [ref 24]. 95% CIs from 2,000 bootstrap resamples.
Analytical methods	12f	E	Describe any model updating (e.g., recalibration) arising from the model evaluation, either overall or for particular sociodemographic groups or settings.	Not applicable — no model updating performed. The need for future Platt scaling or isotonic recalibration before deployment (given RF's calibration slope of 1.369) is discussed in Strengths and limitations.
Analytical methods	12g	E	For model evaluation, describe how the model predictions were calculated (e.g., formula, code, object, application programming interface).	Not applicable — this is a development study. Predictions for the internal test set computed using the trained scikit-learn / XGBoost model objects (predict_proba). Code available upon reasonable request.
Class imbalance	13	D;E	If class imbalance methods were used, state why and how this was done, and any subsequent methods to recalibrate the model or the model predictions.	Methods → Statistical analysis — SMOTE applied exclusively to the training set after the train/test split to address 16.88% event rate, ensuring no synthetic samples leaked into the test set [ref 21]. Calibration was assessed on the unmodified (non-SMOTE) test set. No post-hoc recalibration applied; the resulting RF calibration slope (1.369) and its implications are discussed in Strengths and limitations.
Fairness	14	D;E	Describe any approaches that were used to address model fairness and their rationale.	Formal fairness analyses (e.g., subgroup performance by race/ethnicity, sex,

Section / Topic	Item	Type	Checklist item	Reported in manuscript
				socioeconomic status) were not conducted, as MIMIC-IV provides limited and partially missing sociodemographic granularity beyond age and sex. Sex- and age-stratified analyses are recommended in future external validation studies. This limitation is acknowledged in Strengths and limitations.
Model output	15	D	Specify the output of the prediction model (e.g., probabilities, classification). Provide details and rationale for any classification and how the thresholds were identified.	Methods → Model evaluation / Results → Performance comparison — model outputs predicted probability (continuous, 0–1) of thrombosis. For binary classification, optimal thresholds identified using the Youden index (J = sensitivity + specificity − 1). RF optimal threshold = 0.347 (Youden's J = 0.505), reported alongside default 0.5 threshold (Table 2; Methods → Model evaluation [ref 25]).
Development vs evaluation	16	D;E	Identify any differences between the development and evaluation data in healthcare setting, eligibility criteria, outcome, and predictors.	Results → Study population — development (training) and evaluation (test) data drawn from the same MIMIC-IV cohort via stratified random splitting; identical setting, eligibility criteria, outcome definition, and predictor measurement. Baseline characteristic comparison (Additional file 2) confirmed no statistically significant imbalance across all 29 predictors.
Ethical approval	17	D;E	Name the institutional research board or ethics committee that approved the study and describe the participant-informed consent or the ethics committee waiver of informed consent.	Declarations → Ethics approval and consent to participate — MIMIC-IV approved by IRB of Beth Israel Deaconess Medical Center (approval no. 2001P001699). Informed consent waived owing to retrospective use of de-identified data. This secondary analysis required no additional approval.
**OPEN SCIENCE**				
Funding	18a	D;E	Give the source of funding and the role of the funders for the present study.	Declarations → Funding — funded by Jilin Province Science and Technology Development Plan (YDZJ202502CXJD064), National Clinical Key Specialty Construction Program ([2025] No. 284), Jilin Province Health Plan (2025WS-KA031), and Jilin Province Natural Science Foundation (YDZJ202601ZYTS678). Funders had no role in study design, data analysis, interpretation, or manuscript preparation.
Conflicts of interest	18b	D;E	Declare any conflicts of interest and financial disclosures for all authors.	Declarations → Competing interests — all authors declare no competing interests.
Protocol	18c	D;E	Indicate where the study protocol can be accessed or state that a protocol was not prepared.	No prospective study protocol was prepared, as this is a retrospective secondary analysis of a publicly available database. Analytical plan was

Section / Topic	Item	Type	Checklist item	Reported in manuscript
				specified before primary analyses; deviations during peer review (addition of DCA, multiple imputation, patient-clustered splitting, feature selection experiment, and Youden-based threshold reporting) are documented in this revised manuscript.
Registration	18d	D;E	Provide registration information for the study, including register name and registration number, or state that the study was not registered.	The study was not prospectively registered, as it is a retrospective secondary analysis of routinely collected data not subject to clinical trial registration requirements.
Data sharing	18e	D;E	Provide details of the availability of the study data.	Declarations → Availability of data and materials — MIMIC-IV v3.1 is publicly available at https://physionet.org/content/mimiciv/3.1/ following completion of the required CITI training and signed data use agreement.
Code sharing	18f	D;E	Provide details of the availability of the analytical code.	Declarations → Availability of data and materials — analytical code (Python 3.8.20, scikit-learn 1.3.2, XGBoost 2.1.4, imbalanced-learn 0.12.4) is available from the corresponding author upon reasonable request. The complete environment specification is also provided in Additional file 3.
**PATIENT & PUBLIC INVOLVEMENT**				
Patient & Public Involvement	19	D;E	Provide details of any patient and public involvement during the design, conduct, reporting, interpretation, or dissemination of the study or state no involvement.	No patients or members of the public were directly involved in the design, conduct, reporting, or dissemination of this study. Findings will be communicated to clinicians via peer-reviewed publication and to patient stakeholders through plain-language summaries on institutional channels following acceptance.
**RESULTS**				
Participants	20a	D;E	Describe the flow of participants through the study, including the number of participants with and without the outcome and, if applicable, a summary of the follow-up time. A diagram may be helpful.	Results → Study population / Figure 1 — full participant flow diagram. 14,595 PICC insertions identified; 6,450 (44.2%) excluded for missing predictors; final cohort n = 8,145 PICC insertions from 6,376 unique patients; 1,375 (16.88%) thrombotic events.
Participants	20b	D;E	Report the characteristics overall and, where applicable, for each data source or setting, including the key dates, key predictors (including demographics), treatments received, sample size, number of outcome events, follow-up time, and amount of missing data. A table may be helpful. Report any differences across key demographic groups.	Results → Study population / Table 1 — baseline characteristics for the entire cohort and stratified by thrombosis status. All 29 predictors reported with means ± SD or n (%) and between-group comparisons (Welch's t-test or chi-square test).
Participants	20c	E	For model evaluation, show a comparison with the development data of the distribution of important predictors (demographics, predictors, and outcome).	Results → Study population — training (n = 6,516) and test (n = 1,629) sets compared; no statistically significant imbalance across all 29 predictors and the outcome. Detailed comparison provided in Additional file 2.

Section / Topic	Item	Type	Checklist item	Reported in manuscript
Model development	21	D;E	Specify the number of participants and outcome events in each analysis (e.g., for model development, hyperparameter tuning, model evaluation).	Results → Study population / Table 1 / Sensitivity analyses — Primary analysis: training n = 6,516 (1,100 events); test n = 1,629 (275 events). 5-fold CV: ~1,303 per fold. Patient-clustered split: same totals but partitioned by patient ID. First-PICC-only: n = 6,376 (1,019 events). Multiple imputation: n = 14,595 (m = 5 datasets). Subgroup (no prior thrombosis): n = 1,218 (123 events).
Model specification	22	D	Provide details of the full prediction model (e.g., formula, code, object, application programming interface) to allow predictions in new individuals and to enable third-party evaluation and implementation, including any restrictions to access or re-use (e.g., freely available, proprietary).	Methods → Statistical analysis and machine learning / Additional file 3 — complete hyperparameters reported. The trained Random Forest model object (.pkl) and inference scripts are available from the corresponding author upon reasonable request to enable external evaluation. The model is not currently deployed as a public API.
Model performance	23a	D;E	Report model performance estimates with confidence intervals, including for any key subgroups (e.g., sociodemographic). Consider plots to aid presentation.	Results → Performance comparison / Table 2 / Figures 2, 3, 4 — all metrics with bootstrap 95% CIs (n = 2,000) for all five models. ROC curves with 95% CI bands (Figure 2), calibration curves (Figure 3), decision curves (Figure 4). Subgroup analysis (no prior thrombosis history) reported (Additional file 7).
Model performance	23b	D;E	If examined, report results of any heterogeneity in model performance across clusters.	Results → Sensitivity analyses (patient-clustered splitting) — RF AUC-ROC = 0.696 under patient-level splitting versus 0.809 under random insertion-level splitting, indicating within-patient correlation contributed to primary-analysis discrimination. First-PICC-only analysis: AUC = 0.717.
Model updating	24	E	Report the results from any model updating, including the updated model and subsequent performance.	Not applicable — no model updating performed in this development study.
**DISCUSSION**				
Interpretation	25	D;E	Give an overall interpretation of the main results, including issues of fairness in the context of the objectives and previous studies.	Discussion (paragraphs 1–5) — interpretation of RF's superior discrimination, the dominant role of prior thrombosis history, the clinical paradox of elevated coagulation parameters in thrombosis cases, and findings placed in the context of previous PICC thrombosis prediction studies [refs 7, 17, 19].
Limitations	26	D;E	Discuss any limitations of the study (such as a non-representative sample, sample size, overfitting, missing data) and their effects on any biases, statistical uncertainty, and generalizability.	Strengths and limitations — six explicit limitations: (i) single-center retrospective design; (ii) ICD-based outcome ascertainment underestimating asymptomatic thrombi; (iii) 44.2% complete-case exclusion and potential selection bias; (iv) within-patient correlation from multiple PICCs per patient; (v) absence of catheter-specific variables (gauge, lumens, insertion site); (vi) lack of head-to-head comparison with established clinical risk scores (e.g., Caprini).

Section / Topic	Item	Type	Checklist item	Reported in manuscript
Usability	27a	D	Describe how poor quality or unavailable input data (e.g., predictor values) should be assessed and handled when implementing the prediction model.	Discussion (paragraph on clinical implementation) — the feature selection experiment supports parsimonious 15-feature implementations as a strategy to lower data-completeness barriers (95.5% of full-model AUC retained). Where individual predictor values are missing or older than 24 hours, the system should flag the prediction as provisional and recommend updated testing.
Usability	27b	D	Discuss whether users will be required to interact in the handling of the input data or use of the model, and what level of expertise is required of users.	Discussion (paragraph on clinical implementation) — proposed deployment as an automated EHR-integrated alert triggered at PICC order placement, with no manual data entry required. End users (ICU clinicians) require no specialist statistical or ML expertise; outputs presented as risk probability and Youden-thresholded risk category.
Usability	27c	D;E	Discuss any next steps for future research, with a specific view to applicability and generalizability of the model.	Conclusions — explicit calls for (i) external validation in multi-center datasets, (ii) prospective implementation studies, (iii) inclusion of catheter-specific predictors, and (iv) head-to-head comparison with existing clinical risk scores.

TRIPOD+AI for Abstracts Checklist

The TRIPOD+AI for Abstracts checklist contains 13 essential items for journal or conference abstracts of prediction model studies. The Abstract of the present manuscript is structured to address each item.

Section / Topic	Item	Checklist item	Reported in abstract
Background and objectives	A1	Identify the study as developing or evaluating the performance of a multivariable prediction model. State the study objectives.	Abstract — Background and Methods sentences identify the study as developing/comparing five ML models for predicting PICC-associated thrombotic complications.
Methods – source of data	A2	Describe the key elements of the study setting and source of data.	Abstract → Methods — retrospective cohort study; MIMIC-IV database; ICU setting.
Methods – participants	A3	Describe the eligibility criteria for participants.	Abstract → Methods — adult ICU patients receiving PICC insertion.
Methods – outcome	A4	Specify the outcome being predicted.	Abstract → Methods — PICC-associated thrombotic complications (binary outcome).
Methods – predictors	A5	Specify the key predictors.	Abstract → Methods — 29 predictors across demographics, coagulation, hematologic, vital signs, comorbidities, and treatment.
Methods – sample size	A6	State the sample size and number of outcome events.	Abstract → Methods — 8,145 PICC insertions; 6,376 unique patients; 16.88% event rate.

Section / Topic	Item	Checklist item	Reported in abstract
Methods – statistical analysis	A7	Specify the analytical methods used (and how the model was developed and evaluated).	Abstract → Methods — five ML algorithms; SMOTE on training set only; 5-fold CV grid search; bootstrap CIs; DeLong's test; DCA.
Results – participants	A8	Report the number of participants and outcome events for each analysis.	Abstract → Methods/Results — n = 8,145; 1,375 events.
Results – model performance	A9	Report the predictive performance of the model (with confidence intervals).	Abstract → Results — RF AUC-ROC 0.809 (95% CI 0.780–0.838); DCA net benefit 1–50% threshold range.
Discussion – interpretation	A10	Give an overall interpretation of the results.	Abstract → Conclusions — supports development of ML-based clinical decision support tools for individualized thrombosis risk stratification.
Discussion – limitations	A11	State the main limitations of the study.	Abstract → Results — sensitivity analyses (patient-clustered splitting, MI, first-PICC-only) confirmed robustness; limitations detailed in main text.
Other – funding	A12	Provide the source of funding for the study.	Funding section in Declarations.
Other – registration	A13	Provide the registration number and name of the registry.	Not registered — retrospective secondary analysis. Stated in Item 18d above.

Completed by the corresponding author. Page numbers refer to the revised manuscript text; if the journal renumbers pages during typesetting, this checklist will be updated to reflect the published page numbers.
